# Acute Endovascular Stroke Treatment in Germany in 2019: Results from a Nationwide Database

**DOI:** 10.1007/s00062-020-00989-w

**Published:** 2021-01-22

**Authors:** S. Rohde, W. Weber, A. Berlis, H. Urbach, P. Reimer, P. Schramm, German Society of interventional Radiology and minimal invasive Therapy, German Society of Interventional Radiology and Minimal Invasive Therapy (DeGIR) and the German Society of Neuroradiology (DGNR)

**Affiliations:** 1 Klinik für Radiologie und Neuroradiologie, Klinikum Dortmund gGmbH, Beurhausstr. 40, 44137 Dortmund, Germany; 2 Institut für Diagnostische Radiologie, Neuroradiologie und Nuklearmedizin, Universitätsklinikum Knappschaftskrankenhaus Bochum, Bochum, Germany; 3 Diagnostische und Interventionelle Neuroradiologie, Universitätsklinikum Augsburg, Augsburg, Germany; 4 Klinik für Neuroradiologie, Universitätsklinikum Freiburg, Freiburg, Germany; 5 Diagnostische und Interventionelle Radiologie, Städtisches Klinikum Karlsruhe, Karlsruhe, Germany; 6 Institut für Neuroradiologie, Universitätsklinikum Schleswig-Holstein, Campus Lübeck, Lübeck, Germany

**Keywords:** Thrombectomy, Stroke, Quality assurance, Database, Stroke logistics

## Abstract

**Purpose:**

Since the incidental discovery and systematic introduction of mechanical endovascular stroke treatment in 2015 there are few reports about the real-life situation in daily clinical practice. The aim of this study was to evaluate the mechanical thrombectomy data documented in the quality assurance database of the German Society for Interventional Radiology and Minimally Invasive Therapy (DeGIR) and the German Society of Neuroradiology (DGNR) in 2019.

**Methods:**

We retrospectively analyzed the clinical and procedural data of all mechanical thrombectomies that were entered into the voluntary nationwide database in 2019. The information of each procedure was provided on a standardized web-based data sheet. Data were exported and analyzed by a group of experts on behalf of the DGNR.

**Results:**

A total of 13,840 data sets from 158 participating centers could be analyzed. Mean age of the patients was 74 ± 13 years; 53.9% were female. Vessel occlusion was located in the anterior circulation in 87.4%, in the posterior circulation in 10.7%. On hospital admission, the median National Institutes of Health Stroke Scale (NIHSS) was 14 (lower/upper quartile 10/19); at hospital discharge, median NIHSS had dropped to 9 (lower/upper quartile 2/12; *p* < 0.001).

Recanalization of the occluded vessel segment was successful (TICI 2b + 3) in 88.4%. The reported complication rate was 7.3%, with subarachnoid hemorrhage as the most frequent complication (3.4%), followed by parenchymal hemorrhage (1.7%) and embolization in new territories (1.2%).

Overall, the median time interval from symptom onset to hospital admission was 94 min (quartiles 59/180 min), the median time from hospital admission to groin puncture was 74 min (lower/upper quartile 47/103 min), and the median duration of the procedure 43 min (lower/upper quartile 25.2/73.2 min). A comparison between primary and secondary referral revealed a significant faster symptom-to-intervention time for primary referrals, whereas in-house workflows showed no significant difference.

**Conclusion:**

The analysis represents the largest documented cohort of acute stroke patients treated by thrombectomy. The documentation allows for a detailed evaluation of procedural, clinical, logistic and radiation exposure data and might be used for monitoring the quality of the treatment on a nationwide scale.

## Introduction

In 2012 the German Society for Neuroradiology (DGNR) started a nationwide registry to document the safety and quality of neuroradiological interventions, divided into vessel-opening (module E) and vessel-occluding (module F) procedures of the extracranial and intracranial supra-aortic vasculature. Both modules were embedded into the registry of the German Society for Interventional Radiology and Minimally Invasive Therapy (DeGIR) documenting interventions in general radiology (modules A–D) since 2005.

The main goal of the web-based voluntary national registry is to monitor the number and quality of the different interventional procedures; however, with an increasing number of participating sites all over Germany and solitary centers in Austria and Switzerland [1], the registry offers a close look at real-life data of these procedures and a different perspective in addition to rigorous clinical trial. This is especially true for thrombectomy as the frequency of this procedure increased in a short period of time following the publication of landmark multicenter studies that proved a high effectiveness of endovascular stroke therapy in 2015 [2–8]. The endovascular treatment option thus became an evidence-based treatment for severe ischemic stroke (National Institutes of Health Stroke Scale [NIHSS] ≥ 6 for stroke in the anterior circulation) and has been accordingly recommended in several national and international guidelines [9, 10]. The practice patterns in Germany and Austria are generally in line with the European guidelines for thrombectomy [11], which have been endorsed by the DGNR [12].

The aim of this study was to analyze the mechanical thrombectomy data documented in the German DeGIR/DGNR database in 2019 in order to understand the efficacy of the nationwide acute stroke therapy in large vessel occlusion.

## Methods

### Study Design and Ethical Approval

We retrospectively analyzed the clinical and procedural data of all mechanical thrombectomies that were entered into the voluntary, nationwide database in 2019. Detailed information of each procedure was provided by the participating interventional centers in a web-based format and sent to the DeGIR database using both symmetric and asymmetric encoding techniques to ensure anonymization of the records. Data were exported and analyzed by members of the DeGIR (S.R., W.W., P.S.) on behalf of the DGNR. The study protocol and data analysis were approved by the local ethics committee of the University of Münster (No. 2020-420-f-S).

### Data Extraction and Statistical Analysis

Anonymized records from the database in module E, subcategory “Endovascular Stroke Treatment”, were exported in an Excel chart. Data sheets were analyzed if the treatment had been performed between 1 January 2019 (00:00 h) and 31 December 2019 (24:00 h). Columns with time specifications, e.g. “start of symptoms” or “start of interventions”, were controlled for entry errors and revised in cases of unreasonable or negative time intervals. This may occur when the beginning of symptoms or the treatment was before midnight and the end of the procedure was after midnight. Data exploration was performed by a professional statistician using primarily descriptive statistics for categorical and noncategorical variables. Groups were compared by Kruskal-Wallis rank sum test for general differences. Post hoc tests between groups were done by pairwise Wilcoxon rank sum tests with *p*-values adjusted by the false discovery rate procedure. An alpha level of 0.05 was considered significant.

### Parameters

Table 1 gives an overview about the evaluated parameters which could be assigned to a clinical, logistic and interventional category. In order to keep the data entry as simple and fast as possible, single items were defined as nonobligatory, e.g. ASPECTS (Alberta Stroke Program Early CT Score) on admission. Clinical long-term follow-up is voluntary since follow-up visits are currently not reimbursed and mandatory visits would lead to potential legal conflicts with insurance companies and hospitals. Thus, a mandatory request would lower the motivation of interventional sites to participate in the data collection.

**Table 1:** Overview of clinical, logistical and interventional parameters of the DeGIR database for acute endovascular stroke treatment (module E). Valid and missing/invalid data sets of the respective items

Clinical parameters	Obligatory data entry yes/no	Valid data sets	Missing data
Gender, age	Y	13,840	0
NIHSS on admission	Y	13,840	0
NIHSS at discharge	N	8609	5231 (37.8%)
ASPECTS on admission	N	5129	8711 (62.9%)
Localization of occluded vessel segment	Y	13,840	0
IV-lysis	Y	13,840	0
*Logistic parameters*			
Start of symptoms	Y	13,840	0
Time of hospital admission	Y	13,840	0
Start IV-lysis	N	13,840	0
Start Intervention	Y	13,840	0
End Intervention	Y	13,469	371 (2.7%)
*Interventional parameters*			
No. of passes	Y	13,840	0
TICI score before/after thrombectomy	Y	13,840	0
Extracranial/intracranial stenting	Y	13,840	0
Complications	Y	13,840	0
Fluoroscopy time	Y	13,603	237 (1.7%)
Dose area product	Y	13,608	232 (1.7%)

*NIHSS* National Institutes of Health Stroke Scale, *ASPECTS* Alberta Stroke Program Early CT Score, *IV* intravenous, *TICI* thrombolysis in Cerebral Infarction

Frequencies of variables were expressed as absolute and relative numbers, and mean values with standard deviation. Due to the wide range of time intervals between logistic time points, these values were given as median with quartile deviation.

## Results

### Diagnostic and Clinical Assessment

A total of 13,840 data sets from 158 clinical sites/hospitals could be analyzed, 59 (34.3%) hospitals had entered less than 50 data sets, 72 (45.6%) had entered 51–150, and 27 (17.1%) more than 150 data sets. Mean age of the patients was 74 ± 13 years (median 77 years, lower/upper quartile 66/84), 53.9% were female, and 46.1% were male. Vessel occlusion was located in the anterior circulation in 87.4%, in the posterior circulation in 10.7%, and in multiple localizations in 1.9%. A detailed overview about the localization of occluded vessel segments is given in Table 2. Nomination of multiple occluded vessel segments was allowed. This resulted in relatively high numbers for single locations, e.g. petrous and intradural ICA (internal carotid artery) segments.

**Table 2:** Localization of occluded vessel segments, multiple nominations were allowed

Vessel	*N* (%)
ICA—bifurcation	871 (7.6)
ICA—cervical segment	529 (4.6)
ICA—petrous/cavernous segment	626 (5.5)
ICA—intradural segment	1906 (16.6)
M1	2801 (24.5)
M1 distal segment	881 (7.7)
M2	1388 (12.1)
ACA	540 (4.7)
V1–V3	100 (0.9)
V4	200 (1.8)
BA	1227 (10.7)
PCA	381 (3.3)
Subclavian artery/brachiocephalic trunk	6 (0.05)

*ICA* internal carotid artery, *M1/2* segments of the middle cerebral artery, *ACA* anterior cerebral artery, V1–4 segments of the vertebral artery, *BA* basilar artery, *PCA* posterior cerebral artery

Stratification of the patients was performed with multimodal computed tomography (CT) in the vast majority of the cases: In 95% of the cases stroke imaging was done with cranial CT and CT-angiography. Additional CT perfusion and/or magnetic resonance imaging (MRI) was performed in 49% and 8%, respectively. The ASPECTS was available in only 34% of all data sets, because this variable was not a mandatory field in 2019. Of the patients 44% received IV thrombolysis prior to thrombectomy; in 39.7% of the patients the onset of symptoms was not documented.

**Fig. 1:**
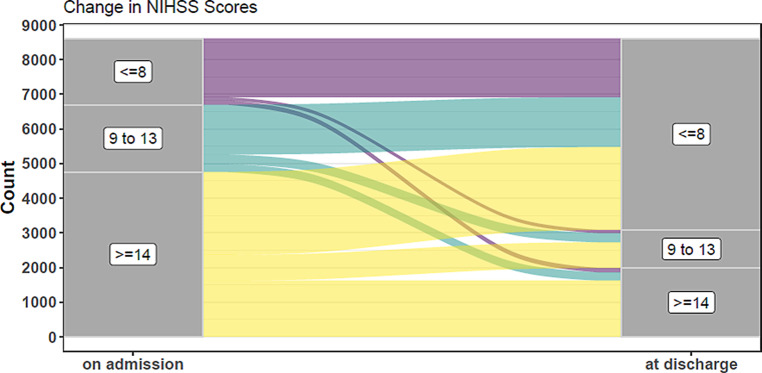
Changes in NIHSS from admission to discharge in 8609 complete data sets

On hospital admission, the median NIHSS was 14 (lower/upper quartile 10/19). At hospital discharge, data were available only in 62% of the cases as this item was not a mandatory field: median NIHSS had dropped to 9 (lower/upper quartile 2/12; *p* < 0.001); 64.2% of the patients had an NIHSS score ≤ 8. Fig. 1 shows the changes in NIHSS in 8609 complete data sets.

### Logistic Parameters

**Fig. 2:**
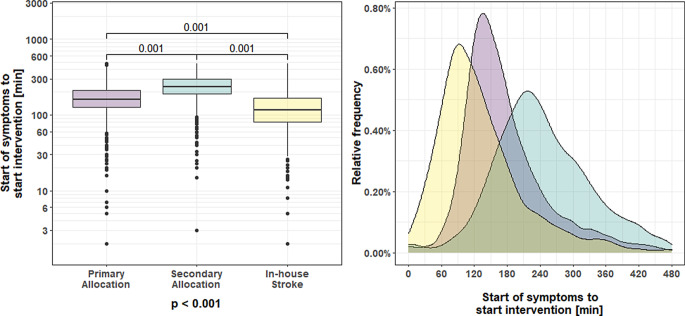
Time intervals from the start of symptoms to the start of the intervention. The figure on the left gives the median ±lower/upper quartile, and range for primary referrals, secondary referrals, and in-house stroke on a logarithmic scale. The figure on the right shows the relative frequency of the three sub-groups on a time scale

Detailed logistic time intervals for primary referral, secondary referral, and in-house stroke are given in Table 3. Overall, the median time interval from symptom onset to hospital admission was 94 min (lower/upper quartile 59/180 min), the time from hospital admission to groin puncture was 74 min (lower/upper quartile 47/103 min). With respect to in-hospital workflow, the overall median time interval from admission to the start of IV-lysis (corresponding to the moment, when imaging diagnostic was finished) was 30 min (lower/upper quartile 19.8/40.2 min), the median time intervals from IV-lysis to the start of the intervention (groin puncture) and the duration of the procedure were 69 min (lower/upper quartile 43.2/120min) and 43 min (lower/upper quartile 25.2/73.2min), respectively. A comparison between primary and secondary referral revealed a significant faster hospital admission-to-intervention time for secondary referrals (median time 79.8min versus 54.0 min, *p* = 0.001); however, that did not compensate for the longer overall time from symptom onset-to-intervention that was about 75 min longer in the secondary compared to the primary referral subgroup (Table 3, Fig. 2). In-house workflows otherwise showed no significant time differences between the subgroups.

**Table 3:** Logistic time intervals by allocation type: primary referral = 8153; secondary referral = 4693; in-house stroke = 994. Data are expressed as median (± quartiles)

	Primary referral	Secondary referral	In-house stroke	*P*^*1*^	*P*^*2*^
Start of symptoms to hospital admission [min]	70.2 (50–113)	180.0 (122–240)	–	0.001	–
Start of symptoms to start IV-lysis [min]	96.0 (75–133)	95.5 (70–135)	60.0 (30–90)	0.225	0.001
Start of symptoms to start intervention [min]	160.2 (125–210)	235.0 (185–3000)	115.0 (80–165)	0.001	0.001
Hospital admission to start IV-lysis [min]	30.0 (20–40)	29.0 (15–41)	–	0.013	–
Hospital admission to start intervention [min]	79.8 (60–105)	54.0 (33–90)	–	0.001	–
Start IV-lysis to start intervention [min]	52.2 (48–74)	130.0 (93–172)	55.0 (33–90)	0.001	0.249
Start intervention to final pass [min]	43.2 (24–71)	43.2 (25–74)	46.0 (26–75)	0.066	0.049

*P*^1^ = Statistical comparison between primary and secondary referral

*P*^2^ = Statistical comparison between primary referral and in-house-stroke

### Interventional Quality Scores and Adverse Events

**Fig. 3:**
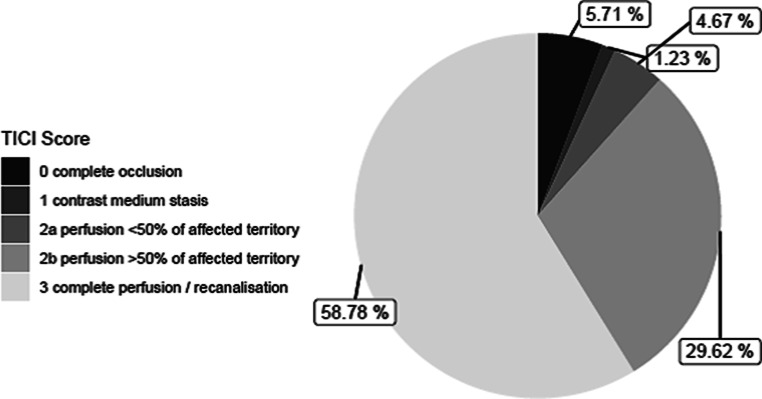
Final TICI score after thrombectomy in 13,840 cases

Fig. 3 shows the distribution of the final TICI score after thrombectomy. A successful recanalization of the occluded vessel segment (TICI 2b + 3) was documented in 88.4%, with a first pass rate of 39.9%. Additional extracranial or intracranial stenting was performed in 8.9% and 3.4%, respectively. A combination of extracranial and intracranial stenting was reported in 0.3% of the cases.

**Fig. 4:**
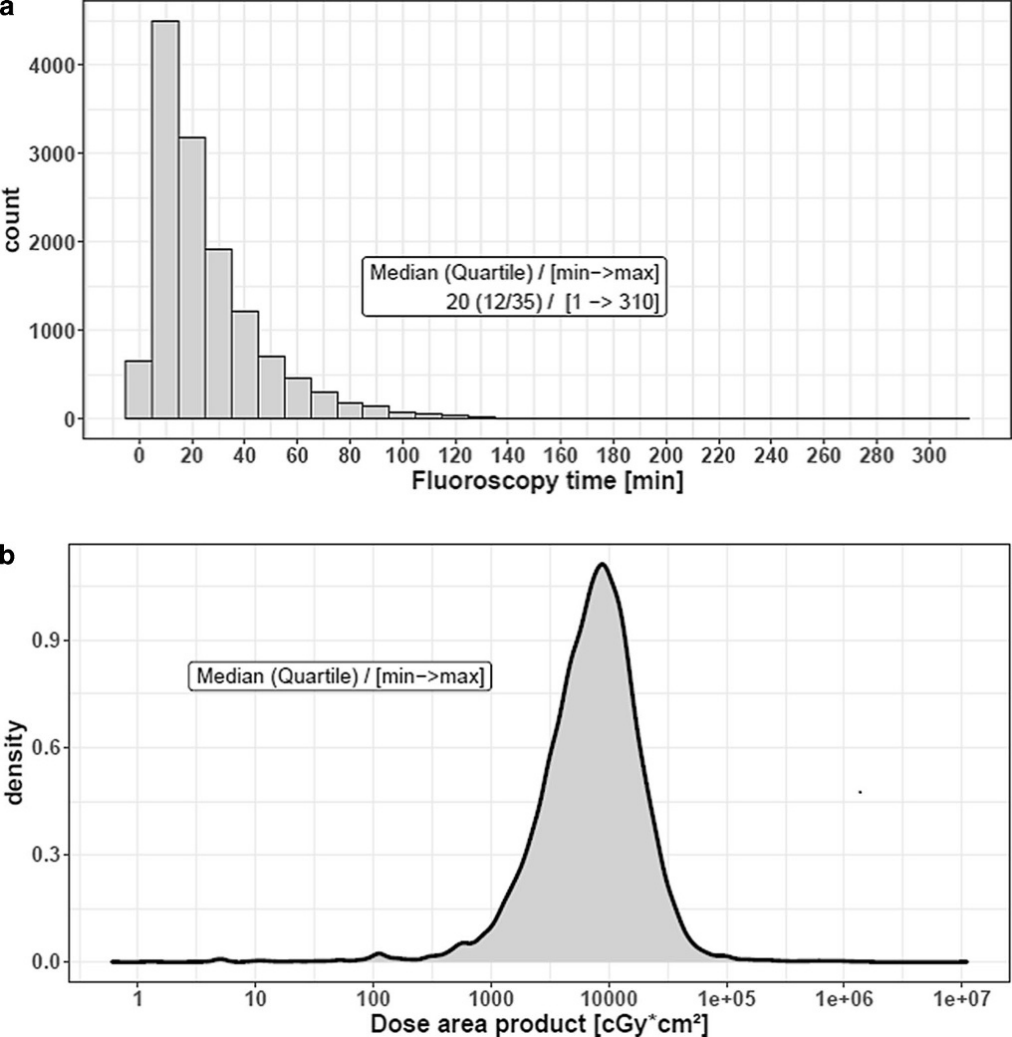
Frequencies of fluoroscopy time (**a**) distribution of the dose area product (**b**) in 13,840 cases

A detailed overview about the frequency of procedure related complications is given in Table 4. Overall, the reported complication rate was 7.3%, with subarachnoid hemorrhage as the most frequent complication (3.4%), followed by parenchymal hemorrhage (1.7%), and extracranial or intracranial vessel dissection (1.6%). Parenchymal and/or subarachnoid hemorrhage were symptomatic in 1.6%. The rate of embolization in new territories was 1.2%. The median fluoroscopy time was 20 min (lower/upper quartile 12/35) with a median dose area product of 7.56 cGy*cm^2^ (lower/upper quartile 4.01/12.89). The frequency of fluoroscopy time and the dose area product is given in Fig. 4.

**Table 4:** Type and absolute/relative frequency of procedure related complications. Multiple entries were possible

	*N* (%)
*Total number of complications*	1008 (7.3)
*Embolization in new territories*	234 (1.2)
*Parenchymal hemorrhage*	263 (1.7)
*Subarachnoid hemorrhage*	465 (3.4)
*Vessel perforation*	181 (1.3)
*Dissection*	
Extracranial	120 (0.9)
Intracranial	107 (0.7)

## Discussion

Since the initial publication of randomized trials on mechanical thrombectomy in 2015, interventional stroke treatment has matured as the treatment of choice in patients with acute occlusion of large cerebral vessels [5]. Since then numerous technical and interventional modifications of the technology were introduced, and enormous efforts have been undertaken both in clinical and scientific settings to advance endovascular techniques, e.g. to address more peripheral occlusion, to define the best interventional setting, or to extend the time-dependent indications [13]. Besides these technical and clinical concerns, a major issue remains the distribution and availability of centers that can provide endovascular stroke treatment 24/7 in order to offer modern stroke service on a nationwide basis; however, there is currently little information about the availability and quality of endovascular stroke treatment in a real-life scenario as a national service approach.

The presented data provide the currently largest and the most coherent information about comprehensive national endovascular stroke treatment in a western country. With nearly 14,000 voluntary documented cases in 2019, the database not only offers detailed clinical and technical information about endovascular stroke treatment, but also gives a depiction about safety aspects, radiation exposure, and—perhaps one of the most important issues—about logistic time intervals for patients with primary and secondary referral.

Compared to the results of the large randomized stroke trials, the reported technical results from our national database are similar. Bush et al. analyzed the pooled data of 1284 patients who were treated in 5 controlled stent-retriever trials (MR CLEAN, ESCAPE, EXTEND-IA, SWIFT PRIME, and REVASCAT) [14]. In contrast to our cohort, patients in the endovascular group (*n* = 634) were younger than in our study (65–71 years versus 74 ± 13 years), had a lower proportion of females (46.4% versus 53.9%), and a somewhat higher mean NIHSS on admission (16–17 versus 14). The lower baseline NIHSS in our cohort might be explained in part by the higher proportion of patients who underwent thrombectomy despite a low NIHSS on admission; 21.1% of the patients in our registry had a baseline NIHSS ≤ 8. This is contrary to randomized controlled trials, where patients with low NIHSS (<8) were mainly excluded [4, 7, 14].

As RCT patients were highly selected (only occlusions in the anterior circulation within a 6–8 h time window were included), the proportion of IV t-PA was higher (83%) compared to our cohort (44%). The relatively low rate of patients that received IV t-PA in our national documentation might be a consequence of a meanwhile extended time window for endovascular stroke treatment; in 39.7% of the patients the symptom onset was unknown or unavailable. Moreover, it might reflect the wider indication for thrombectomy in a real-world scenario, when thrombectomy remains the only treatment option for patients who have contraindications for intravenous lysis, e.g. anticoagulation, recent surgery or known malignancies.

The rate of successful recanalization (TICI 2b or 3) was lower in the meta-analysis (80.4% versus 88.4%). This might be explained in part by the overestimation of the angiographic results in a self-reporting database; however, our results are comparable to the recanalization rates of the EXTEND-IA and the SWIFT-Prime trials with 86.2% and 83%, respectively [4, 7]. In contrast to preceding trials that included several recanalization techniques and devices, only stent-retriever technology and/or aspiration catheters were used for endovascular stroke treatment, resulting in higher recanalization rates than in preceding trials. Procedure-related complications were reported in 6.0–8.3%, mainly embolization into new territories. The reported complication rate in our database was 7.3%.

Thus, gender data and procedural results of our database seem to be reasonable and robust. This applies even more to the presented logistic and fluoroscopic data as these parameters are derived from objective measurements. So far, there is only very little information about such logistic parameters from treatment studies of acute ischemic stroke. Bush et al. reported mean onset-to-groin puncture times (200–269 min) and onset-to-reperfusion times (241–355 min) for the pooled analysis of the 5 RCT. These results are comparable to our data (Table 3), but they do not allow for a detailed evaluation. In contrast, the complete depiction of the logistic process in acute stroke treatment, divided into primary and secondary referral as well as in-house stroke is one of the strongest aspects of the presented data. Our data confirm recent findings of regional registries that direct referral to a comprehensive stroke center leads to better outcome compared to secondary referral in stroke patients [15]. It allows for in-depth analysis of external and in-house process analysis for all kinds of subgroups. For instance, it gives detailed information of logistic time intervals between primary and secondary referrals, e.g. indicating that the mean time from symptom onset to the start of the intervention is about 75 min later in secondary referrals (Table 3). These findings can help in the discussion about stroke logistics in urban and more rural areas and—together with technical parameters like fluoroscopy time and dose—might be used as a helpful quality marker for the participating centers in the future [16].

### Limitations

The main limitation of the presented data is that data entry was not supervised by any means as in randomized controlled trials. Monitoring data entry or checking data entry is beyond the scope of national professional societies. Furthermore, the national health system does not allow for an elegant patient follow-up since digital national records are not available. Thus, the robustness of the data cannot be assessed.

When the specific registry was initiated in 2012 the main goal was to collect clinical and procedural data from all German interventional sites in order to get an almost complete overview of the procedures. As the participation of the sites is voluntary, the number of items is limited with the entry of particular items defined as optional, e.g. the ASPECTS of the initial CT, in order to keep data entry as simple and feasible as possible. Moreover, clinical items such as vascular risk factors and comorbidities are currently not collected. This limits the interpretation of our data, especially when regarding subgroups of indications, logistic parameters or clinical outcome data. From a scientific point of view this concept leads to a loss of robustness and quality of the collected data, especially in comparison to data of controlled clinical studies. To overcome this limitation, the selection of the collected items is monitored and up-dated annually by members of the quality assurance program working group of the DeGIR/DGNR board.

Another limitation is the current lack of long-term follow-up data, especially the clinical outcome at 90 days after treatment. This issue has been discussed intensively when starting the nationwide database and was waived due to practical reasons since follow-up visits are currently not reimbursed; however, as the database is continuously improved, the concern of long-term data might be added in the future, depending on changes within the healthcare system and IT structure.

### Perspective

The continuous development of the nationwide database with its high number of recorded cases bears several areas of interest: The data might even provide a more detailed insight in the quality of endovascular stroke centers and might be used for clinical research on special topics, e.g. the effectiveness and safety of thrombectomy in stroke subgroups, logistic or radiation parameters, or the comparison of high-volume and low-volume centers.

Moreover, it can be used for monitoring the treatment quality, both on a general nationwide scale, when comparing the actual results with preceding years, and on an individual basis for each participating center, when comparing the own results with the nationwide average results and of course the outcomes of randomized controlled trials.
